# Ultrasound-Assisted Enzymatic Hydrolysates from Common Bean and Pumpkin Seed Proteins: Antioxidant and Anti-Inflammatory Properties

**DOI:** 10.3390/antiox15050578

**Published:** 2026-05-03

**Authors:** Erick Huerta-Rodriguez, Omar Sanchez-Jimenez, Cristina Chuck-Hernández, Margarita L. Martinez-Fierro, Idalia Garza-Veloz, Diana L. Cárdenas-Chávez, Cesar A. Ponce-Ponce de Leon, Maria del Refugio Rocha-Pizaña

**Affiliations:** 1 Escuela de Ingenieria y Ciencias, Tecnologico de Monterrey, Av. Eugenio Garza Sada 2501, Monterrey 64849, NL, Mexico; erick.h.rodriguez@tec.mx (E.H.-R.); omar_sanchez@tec.mx (O.S.-J.); cristina.chuck@tec.mx (C.C.-H.); diana.cardenas@tec.mx (D.L.C.-C.); cesar.ponce@tec.mx (C.A.P.-P.d.L.); 2 Molecular Medicine Laboratory, Unidad Academica de Medicina Humana y C.S, Universidad Autonoma de Zacatecas, Carretera Zacatecas-Guadalajara Km 6, Zacatecas 98160, ZAC, Mexico; margaritamf@uaz.edu.mx (M.L.M.-F.); idaliagv@uaz.edu.mx (I.G.-V.)

**Keywords:** bioactive peptides, ultrasound-assisted hydrolysis, plant protein, antioxidant, anti-inflammatory, NF-κB/MAPK, common bean, pumpkin seed

## Abstract

Chronic low-grade inflammation, a key driver of diabetes and fatty liver disease, is present in obesity, which affects 2.1 billion adults as of 2021. Plant-derived bioactive peptides have emerged as promising alternatives to treat inflammation in these pathological processes. This study evaluated the effect of pre- and post-ultrasound-assisted enzymatic hydrolysis on bioactive peptide production and antioxidant activity from common bean (*Phaseolus vulgaris* L.) and pumpkin (*Cucurbita argyoesperma*) seed proteins. Pre-treated hydrolysates were fractionated by molecular weight (<3 kDa and 3–10 kDa) and evaluated for their anti-inflammatory properties by measuring nitric oxide and reactive oxygen species in three treatment schemes (pre-, co-, and post-treatment) in an obesity/inflammatory macrophage model. Ultrasound pre-treatment achieved a higher degree of hydrolysis (peptide production) compared to post-treatment, with corresponding increases in antioxidant activity as measured by the ABTS and ORAC assays. All hydrolysate fractions demonstrated dose-dependent inhibition of pro-inflammatory markers. Fractions administered as a co-treatment showed the strongest anti-inflammatory effect, reducing *Nos-2* and *Cox-2* mRNA expression, as well as secreted levels of pro-inflammatory cytokines (TNF-α, IL-6, MCP-1). These findings indicate that ultrasound treatment, mainly as pre-treatment, represents an effective strategy for producing bioactive peptide hydrolysates with anti-inflammatory properties in vitro that warrant deeper investigation.

## 1. Introduction

Obesity is a multifactorial and complex disease driven by high-caloric diets rich in saturated fats [1], characterized by chronic low-grade inflammation [2]; it is forecasted to affect 3.8 billion adults by 2050 [3]. Individuals with obesity and high-fat diets (HFD) have increased blood lipid levels, especially saturated free fatty acids [1]. Saturated fats disrupt tight junctions in the intestinal epithelium, increasing its permeability; this allows, in turn, the translocation of bacterial endotoxins, such as lipopolysaccharides (LPS), that triggers the inflammatory response via Toll-like receptor (TLR4) agonism [4]. Furthermore, saturated fatty acids promote oxidative stress, leading to cell death, releasing signaling molecules that activate pro-inflammatory pathways and sustain the inflammatory response [5]. Reducing chronic low-grade inflammation in obesity is considered a potential therapeutic strategy to prevent insulin resistance and the progression of other metabolic and cardiovascular diseases [6]. Therefore, there is great interest in developing novel pharmacological and nutritional therapies, as well as in the discovery and utilization of bioactive compounds, to treat inflammation.

Bioactive peptides exhibit an array of physiological effects with health-promoting benefits, including anti-inflammatory and antioxidant properties. These beneficial properties are mediated through different molecular mechanisms, including the suppression of signaling pathways like the nuclear factor-kappa B (NF-κB) and the mitogen-activated protein kinase (MAPK) pathway [5]. These pathways regulate pro-inflammatory cytokine production, M2 macrophage polarization, and the production of pro-resolving mediators which counteract and halt the inflammatory response [7]. Additionally, bioactive peptides can neutralize reactive oxygen (ROS) and nitrogen species (RNS) by directly scavenging radicals and stimulating the intracellular antioxidant response through the nuclear factor erythroid 2-related factor 2 (NRF2) pathway [8].

Advances in peptide structure–activity relationships (SAR) have identified amino acid properties that confer anti-inflammatory and antioxidant bioactivity [8]. Aromatic, basic, and hydrophobic residues contribute to radical scavenging activity [8,9], while peptides containing branched and basic amino acids at terminal positions demonstrate enhanced anti-inflammatory potency through direct NF-κB suppression [9,10,11]. Acidic residues promote metal ion chelation to suppress Fenton-driven ROS generation, synergizing with NRF2-ARE pathway activation [8,12]. Legume hydrolysates contain peptides enriched with these residue types, resulting in downregulation of TNF-α, IL-6, and IL-1β in macrophages alongside enhanced NRF2 activation [8,10,11]. However, the specific SAR and amino acid composition of common bean and pumpkin seed hydrolysates remains uncharacterized, as do the differential effects of processing methods (such as ultrasound-assisted enzymatic hydrolysis) on peptide SAR and bioactivity.

Despite the proven benefits of bioactive peptides, their efficient production has its own challenges. Enzymatic hydrolysis is the most common method to generate peptides; nevertheless, it is limited by low enzymolysis efficiency due to low enzyme–substrate contact, substrate accessibility, long enzymolysis time, and the aggregation of substrates [13]. Emerging processing technologies, such as ultrasound, improve bioactive peptide production efficiency. Ultrasound is a non-thermal technology that uses high-frequency waves (20–100 kHz) that generates a cavitation effect. The cavitation mechanism operates through acoustic bubble collapse, which generates high-pressure and high-temperature microenvironments that disrupt protein–protein interactions [14]. Cavitation unfolds tertiary and secondary structures, disperses aggregates, and exposes hydrophobic and aromatic residues with enhanced scavenging activity [14,15]. These structural modifications increase surface area available for enzymatic cleavage, enhancing the enzyme–substrate contact and enzymolysis efficiency [16].

Common beans (*Phaseolus vulgaris* L.) and pumpkin seeds (*Cucurbita argyosperma*) are the main plant sources of bioactive peptides within the Milpa system, due to their high protein content [17,18]. Common bean cultivar BRSMG Madreperola hydrolysates exhibited anti-inflammatory activity in mice fed an atherogenic diet, reducing tumor necrosis factor α (TNF-α) expression. Furthermore, the hydrolysates also modulated the lipid profile of mice, reducing cholesterol and triglyceride levels [11]. Hydrolysates from Negro 8025 and Pinto Durango cultivars decreased the contents of pro-inflammatory markers nitric oxide (NO) and prostaglandin and reduced the expression of the enzymes responsible for their production in Lipopolysaccharides (LPS)-induced RAW264.7 macrophages. This effect was driven by the suppression of NF-κB translocation [9]. Ultrasound pre-treatment has been demonstrated to increase the degree of hydrolysis (DH) and the antioxidant activity of white kidney, red kidney, and carioca bean cultivar hydrolysates [19,20,21]; nonetheless, other biological activities were not explored.

Pumpkin seed peptides have shown antioxidant, antihypertensive, and antidiabetic properties in vitro [22]. Moreover, administration of pumpkin seed proteins by oral gavage to rats with induced metabolic syndrome resulted in a decrease in serum concentrations of pro-inflammatory markers interleukin 6 (IL-6) and TNF-α [23]. Pumpkin seed hydrolysates generated using ultrasound-assisted enzymolysis exhibited an increase in the hydrolysis rate and the antioxidant capacity, improving their bioactive potential [24]. However, there are no reports comparing the ultrasound pre- and post-treatment effect on the hydrolysis degree and the antioxidant power along with other bioactivities.

There is a lack of studies comparing the benefits of using ultrasound before and after enzymatic hydrolysis. Moreover, the immunomodulatory effects of ultrasound-assisted hydrolysates of bean and pumpkin seed proteins under obesity-associated inflammatory factors (LPS with palmitic acid) have not been reported. This study aimed to generate bioactive peptides from common bean and pumpkin seed proteins using ultrasound-assisted enzymatic hydrolysis and evaluate the impact of pre- and post-treatment of ultrasound on the DH and antioxidant capacity of the hydrolysates. Furthermore, the effect of the hydrolysates on the inflammatory response (NO, ROS, mRNA levels, and cytokine production) was assessed in an obesity/inflammatory model of macrophages induced with LPS and palmitic acid.

## 2. Materials and Methods

### 2.1. Plant Materials and Chemicals

Organic common bean (*Phaseolus vulgaris* L.) cultivar Bayo grains and pumpkin seeds (*Cucurbita argyosperma*) were obtained from local farmers in Tlaxcala, Mexico (19°16′59.2′′ N, 97°56′40.1′′ W). The dry grains were stored under vacuum at 4 °C until use. LPS from *Escherichia coli* O111:B4 were obtained from Sigma-Aldrich (St. Louis, MO, USA). Palmitic acid (PA) from Cayman Chemical (Ann Arbor, MI, USA) was conjugated with fatty acid-free bovine serum albumin (GoldBio, St. Louis, MO, USA) for cellular assays. 2′,7′-dichlorodihydrofluorescein diacetate (DCFH-DA) was purchased from MedChemExpress (Shanghai, China). Unless specified, all chemicals used in the study were of at least analytical grade and were purchased from Sigma-Aldrich (St. Louis, MO, USA).

### 2.2. Protein Extraction from Pumpkin Seeds and Common Beans

Common beans (CBs) and pumpkin seeds (PSs) were milled and sieved with a 0.18 mm mesh. Pumpkin seed flour was defatted using hexane (1:20 *w*/*v*) for 1 h at 60 °C. Proteins were extracted as follows: common bean and pumpkin seed flours were suspended in deionized water (1:10 *w*/*v*), and the pH values were adjusted to 8 and 11 using 1M NaOH, respectively [11,25]. The suspensions were stirred for 1 h at 25 °C, followed by centrifugation at 5000× *g* for 15 min. The pH of the supernatants was adjusted to 4.5 and centrifuged at 10,000× *g* for 15 min. The protein precipitates were lyophilized (FreeZone 4.5 Liter, LABCONCO, Kansas City, MO, USA) at −50 °C and 0.1 mBar for 48 h and stored at –20 °C until use.

### 2.3. Preparation of Protein Hydrolysates and Application of Ultrasound Technology

#### 2.3.1. Gastrointestinal Hydrolysis

Common bean and pumpkin seed protein concentrates (CBP and PSP) were digested using an in vitro gastrointestinal digestion protocol [26]. CBP and PSP were dispersed in water (1:20 *w*/*v*), and the pH was adjusted to 2 using 1M HCl. The dispersions were mixed with pepsin with an enzyme: substrate (E:S) ratio of 1:20 *w*/*w* and incubated at 37 °C with agitation for 2 h. Pepsin was inactivated by adjusting the pH to 7.5. Porcine pancreatin was added to the mixture (E:S 1:20 *w*/*w*) and incubated for another 2 h. The digestion was stopped by heating at 75 °C for 20 min, followed by centrifugation at 16,000× *g* for 10 min. The supernatants of common bean and pumpkin seed protein hydrolysates (CBH and PSH) were lyophilized.

#### 2.3.2. Ultrasound Treatments

Ultrasound was applied as pre-treatment to native protein concentrates (1:20 *w*/*v*) before pepsin and pancreatin addition or as post-treatment to hydrolysates (after pep-sin-pancreatin digestion was finished). The suspensions were sonicated using a 450S-Digital Sonifier (Branson, Brookfield, CT, USA) with a 1/2”diameter disruptor horn, operated at 20 kHz (70% amplitude) for 10 min. These parameters were selected based on the literature precedent for plant protein modification [14,27,28]. The process was carried out in an air-saturated environment. A pulse mode (25 s on and 25 s off) and an ice bath were employed to prevent protein denaturation.

#### 2.3.3. Ultrafiltration

Common bean and pumpkin seed protein hydrolysates pre-treated with ultrasound were ultrafiltered for cell assays. Pierce™ Protein Concentrators PES (Thermo Fisher Scientific, Waltham, MA, USA) of 10K and 3K MWCO and Microsep Advance Centrifugal Devices of 1K MWCO (Pall Corporation, Port Washington, NY, USA) were used to fractionate the hydrolysates. The retained fractions of 3–10 kDa and <3 kDa from common bean (CB10 and CB3) and pumpkin seed (PS10 and PS3) were lyophilized and stored at –20 °C until use.

### 2.4. Degree of Hydrolysis (DH)

The DH of the hydrolysates, with or without ultrasound treatment, was determined employing the o-phthaldialdehyde method of Nielsen et al. [29] without modifications.

### 2.5. Sodium Dodecyl Sulfate Polyacrylamide Gel Electrophoresis (SDS-PAGE)

The molecular weight of proteins from CBP, PSP, and their hydrolysates was characterized using a 12% Tris-Glycine SDS-PAGE under reducing conditions in a Mini-PROTEAN Tetra Cell (Bio-Rad, Hercules, CA, USA) as previously described [30]. Protein concentration was determined using the Bradford method [31], and 20 μg of protein was loaded per well. BlueEasy Prestained Protein Marker (Nippon Genetics Europe, Düren, Germany) was used to estimate the molecular weight of proteins. Gels were stained with Coomassie Brilliant Blue R-250 (Bio-Rad, Hercules, CA, USA).

### 2.6. Tricine–SDS-PAGE

Tricine–SDS-PAGE was used to visualize the molecular weight of peptides from the different hydrolysates. Peptide concentrations were quantified using the Pierce Quantitative Peptide Assays (Thermo Fisher Scientific, Waltham, MA, USA). Samples (20 μg) were loaded in 15% Tris–Tricine gels; electrophoresis was performed in a Mini-PROTEAN Tetra Cell (Bio-Rad, Hercules, CA, USA) following the methodology of Haider et al. [32]. Gels were stained with Coomassie Blue G-250 (Bio-Rad, Hercules, CA, USA). The Spectra Multicolor Low Range Protein Ladder (Thermo Fisher Scientific, Waltham, MA, USA) was used to estimate the molecular weight of peptides.

### 2.7. In Vitro Antioxidant Capacity

The in vitro antioxidant capacity of the hydrolysates was evaluated by the 2,2′-Azino-bis (3-ethylbenzothiazoline-6-sulfonic acid) (ABTS) and oxygen radical antioxidant capacity (ORAC) assays following the protocols described by Re et al. [33] and Ou et al. [34], without modifications, respectively. Trolox standard curves (R^2^ > 0.98) were constructed for the ABTS (25–200 μmol/L) and ORAC (6.25–200 μmol/L) assays. The results were expressed as μmol Trolox equivalents (TE)/g of protein.

### 2.8. Anti-Inflammatory and Antioxidant Properties in Macrophages

#### 2.8.1. Cell Culture

Murine macrophage RAW264.7 cells (American Type Culture Collection, Rockville, MD, USA) were routinely cultured in complete medium DMEM/F12 with 10% FBS (Gibco, Thermo Fisher Scientific, Waltham, MA, USA) and 1% of Penicillin-Streptomycin (10,000 U/mL) maintained at 37 °C with 5% CO_2_ and 95% humidified air. All cellular assays used RAW264.7 cells at passage 10.

#### 2.8.2. Initial Screening: Cell Viability and NO Production

Initial screening assays were performed to select the working concentrations of the hydrolysate fractions. Cell viability was assessed by seeding RAW264.7 cells in complete medium in 96-well plates at a density of 1 ×10^5^ cells/mL and incubating for 24 h. The medium was removed, and the hydrolysates dissolved in complete media were added at different concentrations (32.5 to 500 μg/mL) and incubated for 24 h. Complete medium was used as a control. Then, cell viability was measured with CellTiter 96 Aqueous One Solution (Promega, Madison, WI, USA) following the manufacturer’s instructions. The results were expressed as % cell viability, compared with the control (100%).

NO production was measured in LPS/PA-induced cells [35]. Briefly, cells were seeded in complete medium 96-well plates at a density of 1 × 10^5^ cells/mL and incubated for 24 h. The medium was removed, and the following treatments were added: basal medium (without FBS) was used as a negative control; LPS (100 ng/mL) and PA (100 μM) in basal medium were used as a positive control; the hydrolysate fractions were dissolved in basal medium with LPS and PA. After 24 h, the NO content of the supernatants was analyzed with Griess Reagent System (Promega) as per manufacturer’s instructions. A standard curve of sodium nitrite (1.56–100 μM) was employed, and the results were expressed as μM of nitrite.

#### 2.8.3. Pre-, Co-, and Post-Treatments

**Figure 1:**
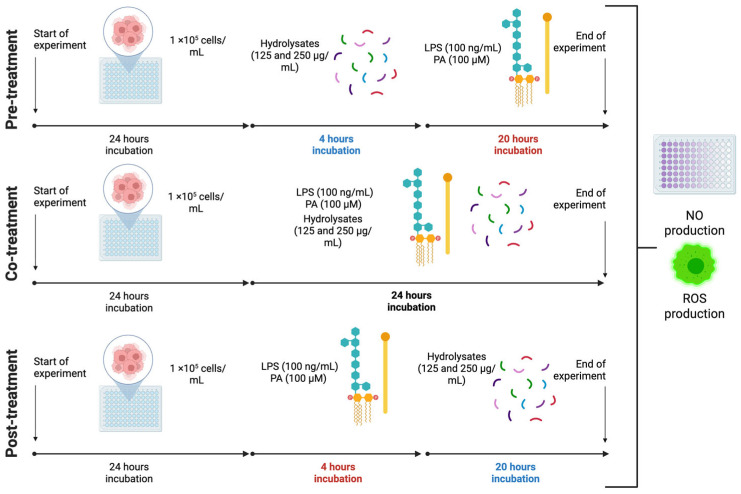
Experimental design of hydrolysate treatment timing and inflammatory response assessment in LPS + PA-stimulated RAW264.7 cells. LPS: lipopolysaccharides, PA: palmitic acid, NO: nitric oxide, ROS: reactive oxygen species. Created in BioRender. Available online: https://BioRender.com/ux33trt (accessed on 16 March 2026).

Different treatment schedules were performed to provide a comprehensive assessment of the activity of the hydrolysate fractions at different stages of the inflammatory response in macrophages. Cells were seeded and treated as described in Section 2.8.2, but with one concentration of each hydrolysate (250 μg/mL). This concentration was selected based on the dose–response data from Section 2.8.2. Following the treatment schemes depicted in Figure 1, NO and ROS production were measured after the treatments.

#### 2.8.4. Intracellular Reactive Oxygen Species (ROS) Production

Cells were seeded and treated as described in Section 2.8.2 and Section 2.8.3. ROS generation was measured as described before [36]. The medium was removed after 24 h of incubation with treatments. Cells were washed in Hank’s balanced salt solution (HBSS), and a solution of DCFH-DA (10 μM) was added and incubated for 30 min at 37 °C. The solution was removed, and the cells were washed twice with HBSS. Finally, 100 μL of HBSS was added to each well, and the fluorescence intensity was measured at excitation and emission wavelengths of 485 and 530 nm, respectively, using a Cytation 5 plate reader (Agilent Biotek, Santa Clara, CA, USA). The results were expressed as % ROS, compared with the negative control (100%).

#### 2.8.5. mRNA Levels of Pro-Inflammatory Enzymes

Following the treatments as in Section 2.8.3 (co-treatments), cells were harvested for total RNA isolation using the RNeasy Plus Mini Kit (Qiagen, Hilden, Germany). The RNA was reverse-transcribed into cDNA following the instructions of the High-Capacity RNA-to-cDNA Kit (Applied Biosystems, Thermo Fisher Scientific, Waltham, MA, USA). Quantitative PCR (qPCR) was conducted using the RealQ Plus 2× Master Mix Green Low ROX (Ampliqon, Odense, Denmark) in a QuantStudio 3 instrument (Applied Biosystems, Thermo Fisher Scientific, Waltham, MA, USA). Mouse-specific primers for inducible nitric oxide synthase (*Nos-2*), cyclooxygenase-2 (*Cox-2*), and peptidylprolyl isomerase B (*Ppib*) are listed in Table 1 and were obtained from [37]. Relative levels of mRNA were calculated using the 2^−ΔΔCT^ method and using *Ppib* as an endogenous control.

#### 2.8.6. Cytokine Production

TNF-α, IL-6, and monocyte chemoattractant protein 1 (MCP-1) production of LPS/PA-induced cells after co-treatment with hydrolysate fractions was measured with ELISA kits. Briefly, cells were seeded in 6-well plates and treated as in Section 2.8.5 with the co-treatments scheme and only one concentration of each hydrolysate (250 μg/mL). Supernatants were collected and centrifuged at 16,000× *g* for 15 min to remove cell debris. Mouse TNF-α (Sigma-Aldrich, St. Louis, MO, USA), Mouse IL-6 SimpleStep, and Mouse MCP1 SimpleStep (Abcam, Boston, MA, USA) ELISA kits were used to quantify the cytokines in the supernatants.

### 2.9. Statistical Analysis

The degree of hydrolysis (DH) and antioxidant capacity (ABTS, ORAC) were determined in triplicate (biological replicates) using three independent enzymatic hydrolysis procedures performed on the bean and pumpkin seed protein concentrates; each biological replicate was measured thrice (technical replicates).

Cellular assays were conducted in biological triplicates using three independent RAW264.7 cell cultures (passage 10), each cultured on separate days to ensure independence. Within each biological replicate, cells were seeded in technical triplicate wells per treatment condition.

Prior to parametric analysis, all datasets were assessed for normality using the Shapiro–Wilk test with significance level α = 0.05; all data met normality requirements. Homogeneity of variance was assessed using Levene’s test with significance level α = 0.05; all datasets met this assumption. Data analysis was conducted using one-way ANOVA with a 95% level of confidence. Differences between treatments were assessed with Tukey’s post hoc test for multiple comparisons (*p* < 0.05). All results were expressed as mean values of the three independent experiments. All data analysis and plots were performed in GraphPad Prism version 10.6.1 (GraphPad Software, Boston, MA, USA) or Minitab version 22.4.0.0 (Minitab Inc., State College, PA, USA).

## 3. Results and Discussion

### 3.1. Effect of Ultrasound Treatment on the Enzymatic Hydrolysis and Antioxidant Activity

**Figure 2:**
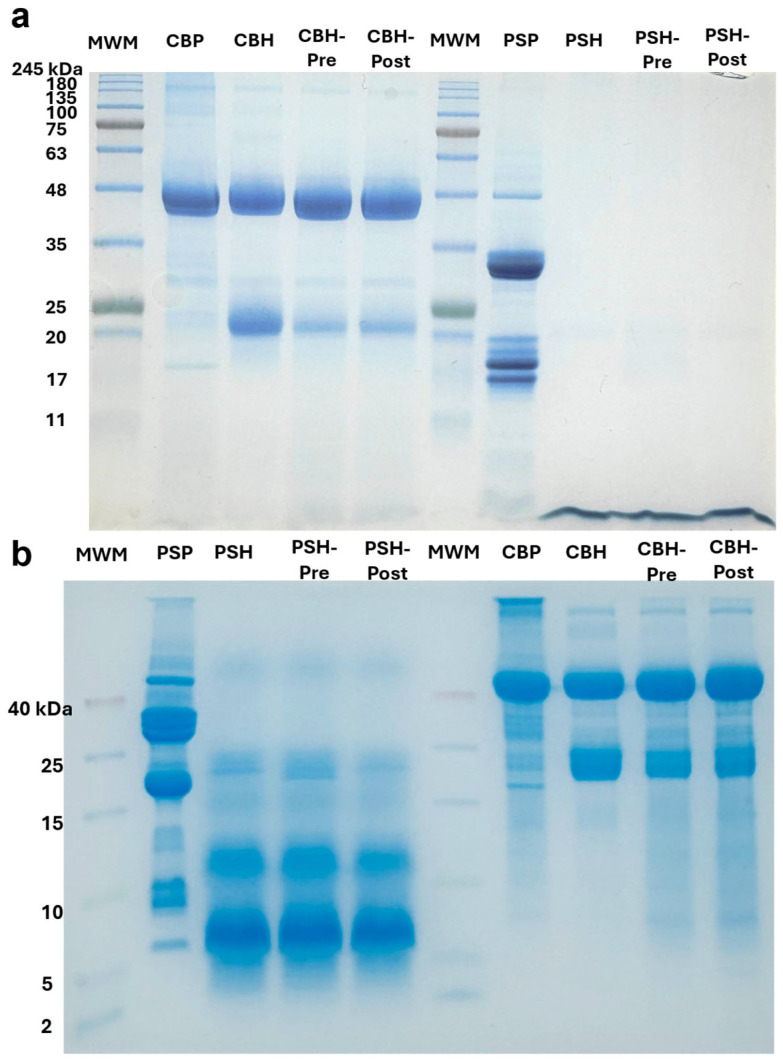
Protein and peptide profiles of common bean and pumpkin seed. (**a**) SDS-PAGE profile of CBP and PSP and their ultrasound-assisted hydrolysates; (**b**) Tricine–SDS-PAGE profiles of CBP and PSP and their ultrasound-assisted hydrolysates. MWM: molecular weight ladder, CBP: common bean protein, CBH: common bean hydrolysate, CBH-Pre: ultrasound pre-treatment of CBH, CBH-Post: ultrasound post-treatment of CBH, PSP: common bean protein, PSH: common bean hydrolysate, PSH-Pre: ultrasound pre-treatment of PSH, PSH-Post: ultrasound post-treatment of PSH.

The gastrointestinal hydrolysis of CBP and PSP with or without ultrasound treatments was visualized by SDS-PAGE and Tricine–SDS-PAGE as shown in Figure 2. CBP showed two intense characteristic bands (35–48 kDa) corresponding to phaseolin (7S) subunits. In CBH, CBH-Pre, and CBH-Post, the phaseolin bands remain intact, indicating ultrasound treatments are not effective in improving the hydrolysis of these subunits (Figure 2a). Similarly, ultrasound bath treatments followed by digestion with Alcalase and Flavourzyme did not increase the hydrolysis of phaseolin [20]. Phaseolins represent 50% of total proteins in common beans and are resistant to gastrointestinal digestion; however, other proteases, such as Alcalase, have been reported to partially hydrolyze these proteins [38]. Although no apparent digestion of phaseolins was initially observed, pre- and post-ultrasound treatments (CBH-Pre and CBH-Post) promoted the appearance of low molecular weight (LMW) bands as shown in the Tricine–SDS-PAGE (Figure 2b), indicating the production of peptides.

PSP showed the characteristic bands of the *Cucurbita* genus at 35 kDA and 14–24 kDa (Figure 2a) corresponding to the storage proteins 11S globulin and 2S albumin [39]. There were no prominent bands above 11 kDa in the SDS-PAGE after digestion with and without ultrasound (PSH, PSH-Pre, and PSH-Post), suggesting PSP is highly digestible. The complete digestion of PSP major bands is consistent with its low anti-nutritional factor content [39,40], which contrasts with CBP, where phaseolin (50% of total protein) resists gastrointestinal hydrolysis [38]. This difference in digestibility is reflected in the markedly higher DH of PSH-Pre (70.87%) compared to CBH-Pre (34.06%), and helps explain the different size–activity effects observed in subsequent assays [39,40]. The Tricine–SDS-PAGE (Figure 2b) confirmed the hydrolysis of the major protein bands and revealed the production of peptides (<10 kDa) and LMW polypeptides below 25 kDa regardless of ultrasound treatment.

The DH is associated with peptide production, where a higher DH is correlated with the presence of smaller peptides with stronger bioactivities [14]. Despite no apparent differences in the hydrolysis of the major storage proteins of common bean, CBH-Pre had a significantly (*p* < 0.05) higher DH compared to CBH and CBH-Post (Table 2). Ultrasound (probe-type) also increased the DH of mung bean (22.28%) and kidney bean (18.29%) [19]. In contrast, ultrasound (bath-type) did not increase the DH of common bean proteins [20]. Nevertheless, this can be explained by the conditions of ultrasound treatment; ultrasound with probe-type sonicators, which interact directly with the sample, achieve a stronger cavitation effect compared to bath-type sonicators, disrupting protein structures and aggregates more efficiently [41].

Ultrasound pre-treatment also significantly increased (*p* < 0.05) the DH compared to post-treatment and PSP control (Table 2). Ultrasound-assisted hydrolysis has been shown to increase the DH of PSH using Neutrase and Flavourzyme with an increase of 65.1% over conventional hydrolysis [24]. Additionally, other works reported lower DH using several proteases, including Alcalase (22.01%) and acid protease (25.61%) [42]. These differences are attributed to the use of different proteases and to the lack of pre-treatment or the use of ultrasonic bath types. Ultrasound pre-treatment disrupts intact, aggregated proteins before enzymatic processing, increasing substrate accessibility for enzyme–protein interaction [41]. In contrast, post-treatment ultrasound acts on already-released peptides where additional structural modification cannot increase enzyme access (since most enzymatic cleavage has already occurred) [41], explaining the additional DH improvement (Table 2).

Antioxidant compounds are of great interest due to their capacity to scavenge free radicals and modulate oxidative stress in pathological conditions, such as chronic inflammation [7]. Antioxidants have two main mechanisms of action, single electron transfer (SET) and hydrogen atom transfer (HAT); therefore, a single assay cannot be used to assess their antioxidant activity [8]. The ABTS (SET mechanism) and ORAC (HAT mechanism) assays showed that both ultrasound pre- and post-treatments increased significantly (*p* < 0.05) the antioxidant capacity of CBH and PSH compared to controls (Table 2). Consistent antioxidant activity increases were observed in beans [14,19], pumpkin seeds [43], wheat [44], and okara seeds [15]. Ultrasound treatment promotes protein unfolding, which facilitates enzyme access and enhances peptide bioactivity. This mechanism seems broadly applicable, regardless of protein composition. Future research should also focus on the evaluation of combined emergent technologies, such as ultrasound coupled with electric pulse fields or high hydrostatic pressure, to increase the yield and bioactivity of food peptides.

The radical scavenging activity of peptides is related to their size; smaller peptides often have stronger antioxidant power due to their low steric hindrance [8]. Additionally, the presence of aromatic, basic, and hydrophobic residues contributes to the overall antioxidant activity [8]. Ultrasound pre-treatments for CBH and PSH exhibited greater antioxidant capacity and DH compared to post-treatments (Table 2). This result suggests that ultrasound induces structural modifications in proteins, changing their secondary structure and aggregation state and increasing the release of small peptides. Furthermore, ultrasound could change the structure of released peptides (as in post-treatments), exposing hydrophobic and aromatic residues, particularly at the N-terminus, with strong scavenging activity [15,45]. Pre-treatment outperformed post-treatment, achieving a higher degree of hydrolysis and antioxidant activity, suggesting ultrasound pre-treatment is an optimal processing strategy for bioactive peptide production. Overall, ultrasound application enhances the production of bioactive peptides and their antioxidant capacity. Nonetheless, optimization of ultrasound parameters (e.g., frequency, amplitude and duration) and industrial scale-up implementation remain necessary to achieve economically viable methods for large-scale bioactive peptide production.

### 3.2. Initial Screening of CBH and PSH Fractions: Cell Viability and NO Production

Based on the results shown in Section 3.1, CBH-Pre and PSH-Pre were separated into 3–10 kDa and <3 kDa fractions for further assays. Cell viability of macrophages was above 95% for all peptide fractions in the range of 32.5 to 500 μg/mL (Supplementary Figure S1).

NO is produced in several cell types in response to various stimuli by three isoforms of NOS. In immune cells, bacterial products (LPS) and saturated fatty acids (PA) can induce the prolonged production of NO, through NOS-2, as part of the inflammatory response and as a mechanism to eliminate pathogens; however, uncontrolled production of NO leads to cell damage [46]. The use of LPS and PA to recreate a model of obesity-induced inflammation in macrophages has been demonstrated; LPS and PA synergistically increased the production of pro-inflammatory cytokines, ROS, and NO [35].

**Figure 3:**
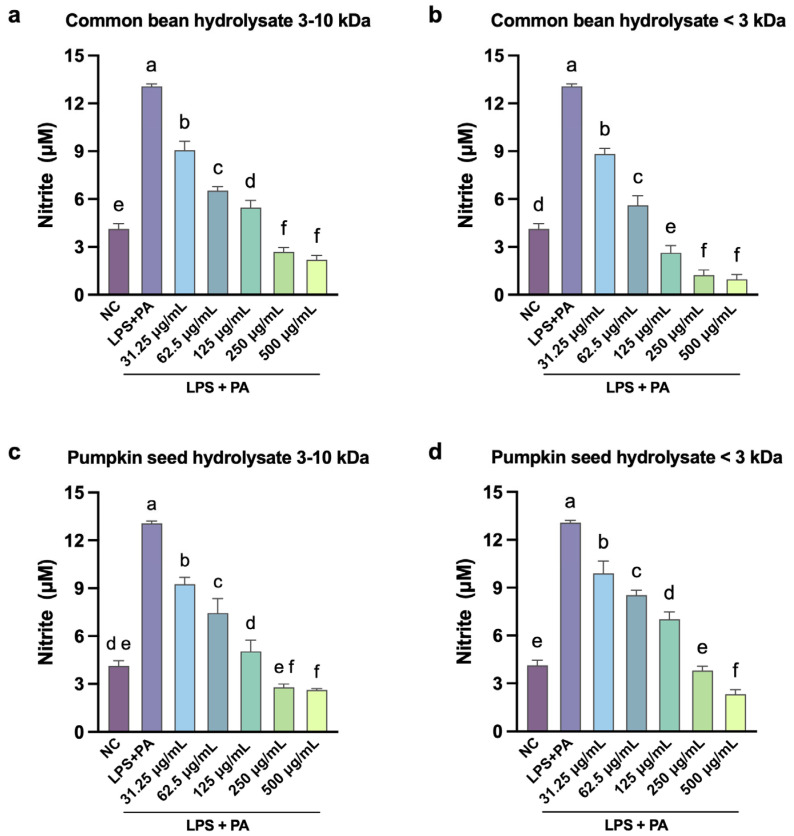
Effect of common bean hydrolysate fractions 3–10 kDa (**a**), < 3 kDa (**b**), pumpkin seed hydrolysate fractions 3–10 kDa (**c**) and < 3 kDa (**d**) on the production of NO in RAW264.7 cells stimulated with LPS and PA. Values are expressed as mean ± standard deviations (*n* = 3). Statistical differences between all treatments were determined by one-way ANOVA followed by Tukey’s post hoc test for multiple comparisons (*p* < 0.05). Bars that do not share a letter (a–f) are significantly different (*p* < 0.05). NC: negative control; PA: palmitic acid; LPS: lipopolysaccharides.

The initial screening of the hydrolysate fractions to assess NO production in macrophages challenged with LPS and PA is shown in Figure 3, where LPS + PA increased 4-fold the production of NO compared to the negative control in accordance with other studies [35,47]. There was a dose-dependent decrease in the production of NO with all hydrolysate fractions (Figure 3). All hydrolysate fractions, at high doses (>250 μg/mL), decreased significantly (*p* < 0.05). NO contents were below negative control levels, indicating a potent anti-inflammatory effect. Dose-dependent NO suppression was observed for all bean and pumpkin fractions. For comparison, foxtail millet [48] reduced NO production by 40% at 250 μg/mL. Soybean hydrolysates [49] achieved 60% NO inhibition at greater concentrations (1000 μg/mL). The dose–response patterns might suggest TLR4 pathway inhibition, possibly through competitive receptor binding or LPS sequestration [50].

Unlike foxtail millet [48] and soybean hydrolysates [49], which reduced NO but remained above the negative control, CB3 suppressed NO production to 4-fold below the negative control. CB3 also surpasses the 52.6% reduction reported for mung bean hydrolysates (250 μg/mL), below 1.45 kDa [10]. This indicates that the common bean < 3 kDa fraction contains potent anti-inflammatory peptides, possibly released from non-phaseolin proteins that are accessible to gastrointestinal enzymes [32].

Notably, for pumpkin seed, the larger fraction (3–10 kDa) outperformed the smaller PS3 in NO reduction, the inverse of the pattern seen for the common bean. This divergence is likely explained by differences in protein composition and digestibility between the two sources. PSP is extensively digested (DH = 70.87%) [39,40], producing a plethora of small peptides across both fractions. In contrast, CBP resists hydrolysis (DH = 34.06%), with phaseolin remaining largely intact [9]. For pumpkin seed, the higher DH means the < 3 kDa fraction likely contains over-digested peptides that have lost bioactive sequences [8,51]. The PS10 peptides may retain the sequence length needed for effective LPS binding or TLR4 interaction, consistent with the known activity of immunomodulatory peptides such as lunasin (5.5 kDa) [52] and DEFB126(1–39)-TP5 (5.2 kDa) [53].

The initial NO dose–response experiments (Figure 3) established that CB10, CB3 and PS10 hydrolysate fractions achieved plateau at 250 μg/mL, with no significant improvement at 500 μg/mL (*p* > 0.05). This plateau effect is consistent with saturation kinetics observed in bioactive peptide studies [54], where increasing concentration beyond the point of maximum biological response produces no additional gains. Accordingly, subsequent assays employed the optimized concentration of 250 μg/mL.

### 3.3. Pre-, Co-, and Post-Treatments of CBH and PSH Fractions: NO and ROS Production

**Figure 4:**
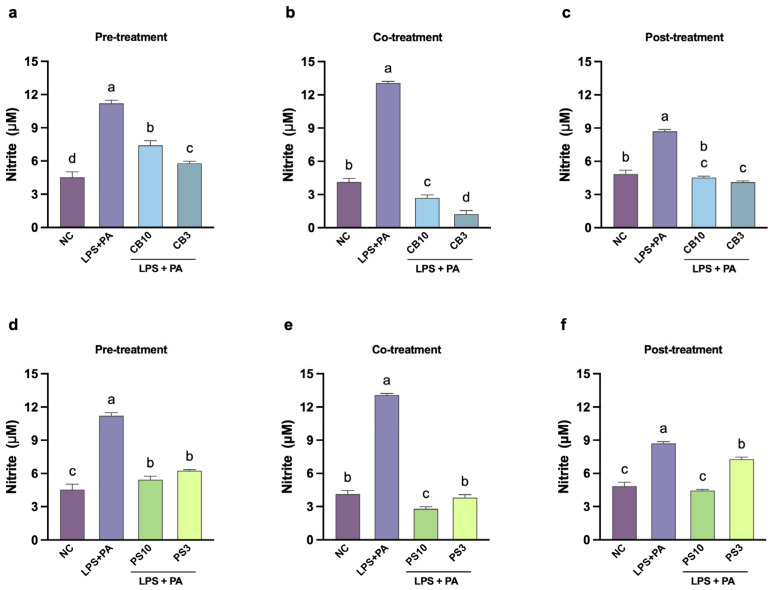
Effect of common bean (**a**–**c**) and pumpkin seed (**d**–**f**) hydrolysate fractions (250 μg/mL) on the production of NO in RAW264.7 cells stimulated with LPS and PA during different treatment schedules. Values are expressed as mean ± standard deviations (*n* = 3). Statistical differences between all treatments were determined by one-way ANOVA followed by Tukey’s post hoc test for multiple comparisons (*p* < 0.05). Bars that do not share a letter (a–d) are significantly different (*p* < 0.05). NC: negative control; PA: palmitic acid; LPS: lipopolysaccharides; CB10: common bean 3–10 kDa fraction; CB3: common bean < 3 kDa fraction; PS10: pumpkin seed 3–10 kDa fraction; PS3: pumpkin seed < 3 kDa fraction.

**Figure 5:**
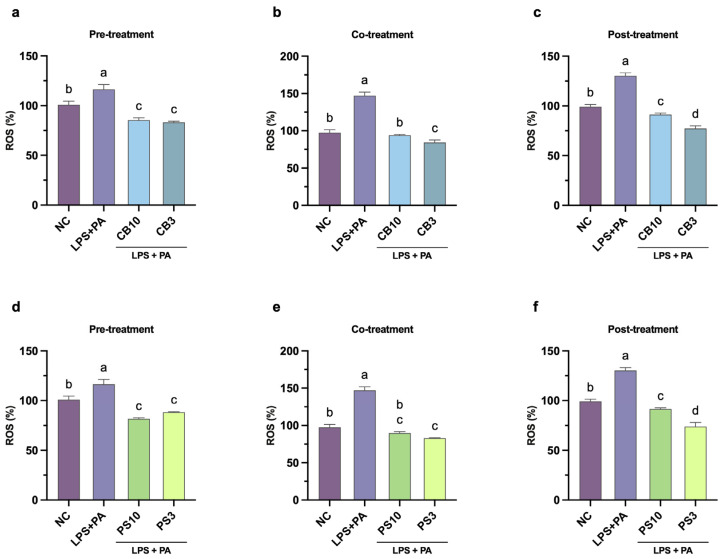
Effect of common bean (**a**–**c**) and pumpkin seed (**d**–**f**) hydrolysate fractions (250 μg/mL) on the production of ROS in RAW264.7 cells stimulated with LPS and PA during different treatment schedules. Values are expressed as mean ± standard deviations (*n* = 3). Statistical differences between all treatments were determined by one-way ANOVA followed by Tukey’s post hoc test for multiple comparisons (*p* < 0.05). Bars that do not share a letter (a–d) are significantly different (*p* < 0.05). NC: negative control; PA: palmitic acid; LPS: lipopolysaccharides; CB10: common bean 3–10 kDa fraction; CB3: common bean <3 kDa fraction; PS10: pumpkin seed 3–10 kDa fraction; PS3: pumpkin seed <3 kDa fraction.

High and sustained levels of NO result in the production of ROS and RNS, causing mitochondrial damage, cell death, and maintaining the inflammatory response. The immunomodulatory effect of the hydrolysate fractions was evaluated under three treatment schemes: pre-, co-, and post-exposure to the LPS and PA challenge (Figure 1). This design allows for assessing the peptide activities in three contexts: protection before an inflammatory trigger (preventive approach), interfering with the signaling pathways during the acute inflammatory phase (onset approach), or promoting the resolution of the inflammatory process (resolution approach) [55]. The effect of the hydrolysate fractions at different stages of the inflammatory (NO) and oxidative stress (ROS) response in macrophages is shown in Figure 4 and Figure 5, respectively. LPS and PA induced significant increments of NO and ROS compared to the negative control across all stages; the maximal NO and ROS responses were observed during co-treatment (13.07 ± 0.15 μM and 147.13 ± 4.72%).

Protein hydrolysates contain a myriad of peptides with different mechanisms of action involved in all stages of inflammation. As expected, hydrolysate treatments, regardless of the fraction and the scheme, significantly (*p* < 0.05) inhibited the production of NO and ROS when compared to the positive control (Figure 4 and Figure 5). During pre-treatment, CB3 and PS10 showed the strongest reduction in NO production, while there was no difference in ROS content among hydrolysate treatments. Possible mechanisms at this stage include competitive binding to the TLR4 receptor, displacing LPS and preventing downstream signaling. This mode of action was demonstrated in silico for the peptides YPFPGPIH [56] and DEFB126 (1–39)-TP5 [53]. Also, cell-permeable peptides may intracellularly block the TL4R, preventing receptor homodimerization and interaction with adapter proteins (TRAM/TIRAP), disrupting the signaling cascade [57]. Future experiments (TLR4 competitive binding assays) are necessary to determine if the hydrolysate fractions operate through either mechanism.

Particularly during co-treatment, all fractions reduced the inflammatory response below the negative control; CB3 showed the strongest reduction in NO and ROS production. The CB3 fraction showed 30% ROS reduction, which is below the 50% reduction in soybean hydrolysates [49,58]. Some of the possible mechanisms at this stage are direct LPS binding and ROS scavenging. For example, cationic peptides from rice bran and soybean have shown LPS-neutralizing activities (assessed with the Limulus amebocyte lysate assay); the cationic peptides bind tightly to the negatively charged LPS, preventing TLR4 activation [50,59]. CBH and PSH may share this mechanism; nonetheless, amino acid sequencing is needed to determine their charge. Additionally, peptides can be transported inside the cells where they interact directly with ROS. The scavenging capacity depends on the amino acid composition of the peptides; however, smaller peptides can easily cross the cell membrane [8]. The stronger ROS suppression (Figure 4 and Figure 5) by CB3 and PS3 (70–80%) compared to larger fractions indicates that smaller peptides are more effective for intracellular ROS scavenging. Conversely, PS10 showed stronger NO suppression, suggesting size-dependent differences in molecular mechanisms.

Finally, all fractions except for PS3 reduced NO content to the levels of negative control as post-treatments. Moreover, all fractions suppressed ROS production significantly (*p* < 0.05) below the negative control levels. Post-treatment NO and ROS suppression was observed, consistent with YPFPGPIH [56] and DEFB126(1–39)-TP5 [53] peptides. This indicates peptides can act on established inflammatory responses through NOS-2 inhibition or ROS scavenging [8,10,50]. Another possible mechanism involves NRF2 pathway activation; low molecular weight, hydrophobic N-terminal and hydrophilic C-terminal peptides have been shown to upregulate antioxidant enzymes (glutathione, catalase, and superoxide dismutase), reducing intracellular ROS [60]. Similarly, the observed suppression of NO production is consistent with inhibition of NF-κB/MAPK signaling, as demonstrated for jack bean [54] and selenium-enriched brown rice peptides [61]. Overall, the concordance between chemical radical scavenging capacity (ABTS and ORAC) and cellular ROS suppression demonstrates antioxidant efficacy at both the chemically and physiologically relevant cellular levels. However, whether CB3 and PS10 act through NRF2 and MAPK pathways requires confirmation by Western blot and reporter assays [54] combined with peptide sequencing and molecular docking analysis.

### 3.4. mRNA Levels of Pro-Inflammatory Enzymes

COX-2 and NOS-2 are key inducible enzymes responsible for the catalysis of pro-inflammatory molecules, prostaglandin E2 and NO, respectively; both enzymes are regulated by NF-κB at the transcriptional level [62]. NF-κB proteins are a family of inducible transcriptional factors that are sequestered and inactive in the cytoplasm through association with IκB inhibitory proteins. LPS and saturated fatty acids bind to immune receptors, such as TLRs, and activate signaling cascades that converge in the phosphorylation of IκB and NF-κB subunits [63]. Phosphorylated IκB liberates NF-κB for activation and nuclear translocation [63]. The continuous activation of NF-κB, induced by exogenous (fatty acids and LPS) and endogenous (oxidative stress), leads to the overexpression of inflammatory enzymes and factors contributing to cell dysfunction, insulin resistance, and progression of metabolic diseases [64].

**Figure 6:**
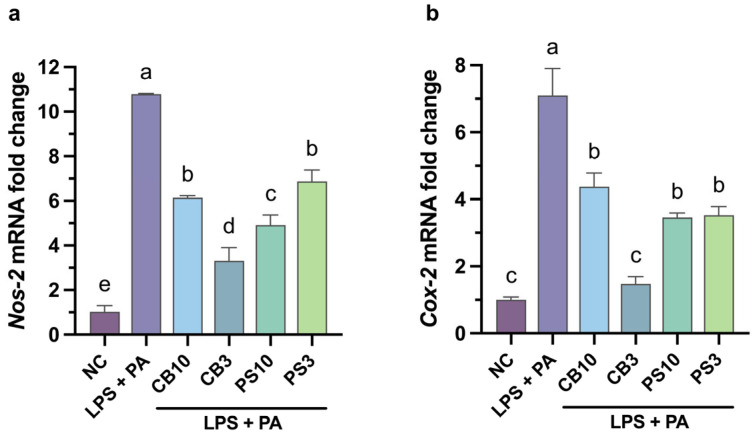
Effect of common bean and pumpkin seed hydrolysate fractions (250 μg/mL) on the expression of *Nos-2* (**a**) and *Cox-2* (**b**) in RAW264.7 cells stimulated with LPS and PA. Values are expressed as mean ± standard deviations (*n* = 3). Statistical differences between all treatments were determined by one-way ANOVA followed by Tukey’s post hoc test for multiple comparisons (*p* < 0.05). Bars that do not share a letter (a–e) are significantly different (*p* < 0.05). NC: negative control; PA: palmitic acid; CB10: common bean 3–10 kDa fraction; CB3: common bean < 3 kDa fraction; PS10: pumpkin seed 3–10 kDa fraction; PS3: pumpkin seed < 3 kDa fraction.

Based on the results shown in Section 3.3, the co-treatment scheme was assessed in the following assays. Stimulation of RAW264.7 macrophages with LPS and PA leads to robust expression of *Nos-2* (10.5-fold) and *Cox-2* (7-fold) mRNA levels compared to unstimulated cells (Figure 6).

Treatment with common bean and pumpkin seed hydrolysate fractions suppressed both *Nos-2* and *Cox-2* mRNA expression (Figure 6), with the CB3 fraction showing the strongest effect again. CB3 reduced *Nos-2* and *Cox-2* expression by over 70%. The CB10, PS10, and PS3 fractions showed moderate suppression; nonetheless, they reduced *Nos-2* and *Cox-2* expression by more than 35%. *Nos-2* and *Cox-2* mRNA downregulation across all treatments indicates that NO reduction involves transcriptional suppression, suggesting NF-κB pathway interference.

The results presented in Figure 6 suggest that peptides from common bean and pumpkin seed proteins inhibit the expression of NF-κB downstream targets, possibly due to the inhibition of NF-κB translocation. Indeed, this type of activity has been reported for some peptides from mung bean protein that have the capability to reduce IκBα phosphorylation and prevent NF-κB activation [10]. Moreover, peptides from jack bean and selenium-enriched brown rice reduced the phosphorylation of p65, an NF-κB subunit [54,61]. Nonetheless, it remains to be determined whether suppression occurs through direct inhibition of upstream kinases (MAPK phosphorylation) or IκB degradation; Western blot analysis of phospho-IκB, phospho-p38, and phospho-ERK would provide direct evidence. Additionally, NF-κB and NRF2 nucleus translocation studies via immunofluorescence assays would confirm transcription factor activation.

### 3.5. Cytokine Production

**Figure 7:**
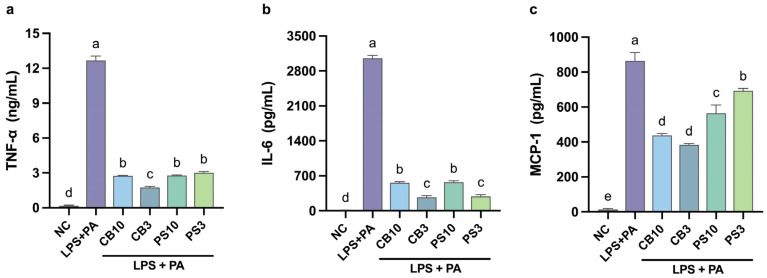
Effect of common bean and pumpkin seed hydrolysate fractions (250 μg/mL) on the production of cytokines TNF-α (**a**), IL-6 (**b**), and MCP-1 (**c**) in RAW264.7 cells stimulated with LPS and PA. Values are expressed as mean ± standard deviations (*n* = 3). Statistical differences between all treatments were determined by one-way ANOVA followed by Tukey’s post hoc test for multiple comparisons (*p* < 0.05). Bars that do not share a letter (a–e) are significantly different (*p* < 0.05). NC: negative control; PA: palmitic acid; CB10: common bean 3–10 kDa fraction; CB3: common bean < 3 kDa fraction; PS10: pumpkin seed 3–10 kDa fraction; PS3: pumpkin seed < 3 kDa fraction.

Cytokines are small signaling proteins that mediate intercellular communication, mainly between the immune system. They have pleiotropic and contradictory effects in different cell types; however, they can be classified as pro-inflammatory or as anti-inflammatory [65]. Pro-inflammatory cytokines are essential to initiate the immune response against infections, but their sustained expression leads to chronic inflammation and tissue malfunction [64]. TNF-α exerts its biologic functions by binding to its cognate surface receptors, initiating the downstream signaling pathways c-Jun N-terminal kinase (JNK) and NF-κB in different cell types. Nevertheless, overexpression can lead to insulin resistance in muscle cells, endothelial cell dysfunction, and augmented lipolysis in adipocytes, leading to cardiovascular issues [66]. The results indicated that all hydrolysate fractions significantly reduced (*p* < 0.05) the expression of TNF-α compared to the positive control (Figure 7a); CB3 showed the greatest reduction (75%). Lower effects were reported with other legumes; TNF-α expression was suppressed by 50% via inhibition of NF-κB following treatment with soybean [67] and jack bean hydrolysates at 500 μg/mL [54]. This suggests anti-inflammatory peptides may share common features, such as basic and hydrophobic residues, that regulate TNF-α expression through NF-κB [61].

IL-6 shows both anti- and pro-inflammatory effects. Elevated levels of IL-6 disrupt the gut barrier, enhancing intestinal permeability [4]. Additionally, it activates the signal transduction and transcription activator 3 (STAT3) pathway and the NF-κB pathway in hepatocytes and muscle cells, leading to steatosis and insulin resistance, respectively [64,68]. The results showed that all hydrolysate fractions decreased IL-6 levels by over 5-fold (Figure 7b), and CB3 had the strongest effect. Lower effects were demonstrated by mung bean and selenium-enriched brown rice hydrolysates at similar concentrations (250 and 100 μg/mL), which reduced IL-6 levels by 48.4% [10] and 40% [61], respectively. Notably, peptides below 3 kDa from Sacha Inchi upregulated the expression of IL-6 in stimulated macrophages but not in basal conditions [58]. However, peptides can have contrasting effects, which are not only related to their molecular weight, but also to their composition and structure.

MCP-1 is a main chemokine, part of the chemoattractant cytokine family, whose main role is to coordinate immune cell recruitment to different sites [69]. This chemokine is expressed by macrophages and other cells (e.g., adipocytes) in patients with metabolic syndrome and associated conditions [64]. Overproduction of MCP-1 by resident macrophages in muscle, liver, and adipose tissues promotes monocyte infiltration, perpetuating the inflammatory process, leading to insulin resistance, cellular dysfunction, and fibrosis [69]. All fractions attenuated MCP-1 production (Figure 7c); CB3 reduced MCP-1 by 2-fold, whereas soy hydrolysates (4 mg/mL) reduced MCP-1 secretion by 30% [70].

TNF-α, IL-6, and MCP-1 were all suppressed simultaneously (Figure 7). This parallel reduction suggests an NF-κB-driven transcriptional suppression rather than independent cytokine inhibition. Notably, CB3 consistently demonstrated stronger anti-inflammatory effects across all three cytokines measured, while PS3 showed variable effects; this suggests that factors other than molecular weight influence the anti-inflammatory activity. For example, soy hydrolysates generated with different enzymes reduced MCP-1 secretion in stimulated macrophages; the peptides with branched and basic amino acids at the C and N terminal showed the strongest effect despite their size [70]. Similarly, peptides from hemp seed [71] and jack bean [54], both with basic and hydrophobic residues, have enhanced anti-inflammatory activities and reduced NF-κB phosphorylation. Nevertheless, the exact role of different residues remains ambiguous and requires further research.

Several mechanistic limitations of this study should be noted. The participation of the NF- κB/MAPK and NRF2 pathways is inferred from downstream endpoints (NO, ROS, mRNA levels and cytokine production) and from precedent in analogous hydrolysates [9,10,54,61]. Direct evidence from Western blot quantification of phosphorylation, NF-κB reporter gene assays and NRF2 nuclear translocation assays was not obtained. Finally, the identity of the bioactive sequences within the hydrolysates remains unknown. Future studies should combine LC-MS/MS peptide sequencing with molecular docking and experimental validation (Western blot of NF-κB/MAPK phosphorylation targets and NRF2 nuclear translocation assays) to establish structure–activity relationships and confirm the mechanistic pathways inferred.

## 4. Conclusions

This study demonstrates that ultrasound-assisted enzymatic hydrolysis, applied before or after hydrolysis, enhances the production of bioactive peptides from common bean and pumpkin seed proteins. Ultrasound pre-treatment was superior to post-treatment, resulting in a higher degree of hydrolysis and antioxidant activity (ABTS and ORAC) across both plant sources. This study demonstrates that ultrasound can disrupt protein tertiary structures and enhance substrate accessibility before enzymatic digestion, increasing enzyme–protein interactions and the release of bioactive peptides. Furthermore, it supports the ultrasound pre-treatment enzymatic hydrolysis as an attractive processing strategy for bioactive peptide production.

Additionally, pre-treated hydrolysate fractions dose-dependently suppressed inflammatory mediator production (NO and ROS) in the three different treatment schemes. Particularly, the CB3 fraction, as co-treatment, presented the most pronounced anti-inflammatory effects in macrophages at mRNA and protein levels. All fractions inhibited secretion of pro-inflammatory cytokines TNF-α, IL-6, and MCP-1. The observed downregulation of *Nos-2* and *Cox-2* mRNA may indicate possible suppression of NF-κB/MAPK signaling. Direct pathway confirmation will require further validation through Western blot analysis of phosphorylated signaling proteins, NF-κB reporter assays to measure transcription factor activity and NRF2 nuclear translocation assays. Future studies must prioritize peptide sequencing via LC-MS/MS to identify the specific bioactive sequences in CB3, followed by structure–activity relationship analysis and molecular docking.

Translation of these in vitro findings to therapeutic application requires assessment of gastrointestinal stability and oral bioavailability. Critical parameters include peptide resistance to gastrointestinal digestion, intestinal permeability, plasma stability, and the achievability of therapeutic concentrations through dietary consumption. Studies employing INFOGEST simulated digestion, Caco-2 intestinal transport models, and pharmacokinetic profiling in animal models are necessary to determine whether these peptides can maintain bioactivity following oral administration and achieve concentrations equivalent to the effective in vitro dose. Encapsulation strategies should also be explored to protect larger peptides and enhance systemic delivery.

Overall, these plant-derived bioactive peptides represent a valuable alternative for developing anti-inflammatory agents to address the growing prevalence of low-grade chronic inflammatory pathologies.

## Figures and Tables

**Table 1:** qPCR primers used in this study.

Gene Name	GeneBank Accession Number	Forward Primer (5′ → 3′)	Reward Primer (5′ → 3′)
*Ppib*	NM_011149.2	AAACAGCAAGTTCCATCGTGTCAT	GAAGCGCTCACCATAGATGCTCT
*Nos-2*	NM_010927.4	GAGCGAGGAGCAGGTGGAA	CCATAGGAAAAGACTGCACCGA
*Cox-2*	NM_011198.5	TCCCTGAAGCCGTACACATCA	TGGACGAGGTTTTTCCACCA

Mouse specific primers were obtained from [37].

**Table 2:** Degree of hydrolysis and antioxidant properties of common bean and pumpkin seed protein hydrolysates.

	Common Bean Hydrolysate		
	Control	Pre	Post
DH (%)	28.64 ± 1.78 ^b^	34.06 ± 0.68 ^a^	30.13 ± 0.93 ^b^
ABTS (μmol TE/g)	203.60 ± 4.69 ^c^	723.00 ± 9.91 ^a^	615.90 ± 7.24 ^b^
ORAC (μmol TE/g)	406.40 ± 11.50 ^c^	1086.00 ± 50.82 ^a^	756.20 ± 42.22 ^b^
	**Pumpkin Seed Hydrolysate**		
	Control	Pre	Post
DH (%)	63.06 ± 1.39 ^b^	70.87 ± 2.13 ^a^	64.58 ± 1.81 ^b^
ABTS (μmol TE/g)	515.80 ± 3.72 ^b^	561.60 ± 10.24 ^a^	554.60 ± 4.77 ^a^
ORAC (μmol TE/g)	2178.00 ± 43.96 ^b^	2450.00 ± 61.46 ^a^	2219.00 ± 70.91 ^b^

Values are expressed as mean ± standard deviations (*n* = 3). Statistical differences between all groups were determined by one-way ANOVA followed by Tukey’s post hoc test for multiple comparisons (*p* < 0.05). Different letter superscripts (a–c) in the same row indicate significant differences among treatments (*p* < 0.05). Control: digestion without ultrasound treatment; DH: degree of hydrolysis; ABTS: 2,2′-azinobis (3-ethylbenzothiazoline-6-sulfonic acid); ORAC: oxygen radical absorbance capacity; TE: Trolox equivalents.

## Data Availability

The original contributions presented in this study are included in the article/Supplementary Materials. Further inquiries can be directed to the corresponding author.

## Supplementary Materials

The following supporting information can be downloaded at: https://www.mdpi.com/article/10.3390/antiox15050578/s1. Figure S1: Effect of common bean hydrolysate fractions 3–10 kDa (**a**), <3 kDa (**b**), pumpkin seed hydrolysate fractions 3–10 kDa (**c**) and <3 kDa (**d**) fractions on cell viability of RAW264.7 cells. Values are expressed as mean ± standard deviations (*n* = 3). Bars that do not share a letter (a) are significantly different (*p* < 0.05).
