# Supplementary figures and images for: Ultrasound-Assisted Enzymatic Hydrolysates from Common Bean and Pumpkin Seed Proteins: Antioxidant and Anti-Inflammatory Properties
Source: Antioxidants (Basel). 2026 May 3;15(5):578. doi: 10.3390/antiox15050578 (PMC13203739; doi:10.3390/antiox15050578)

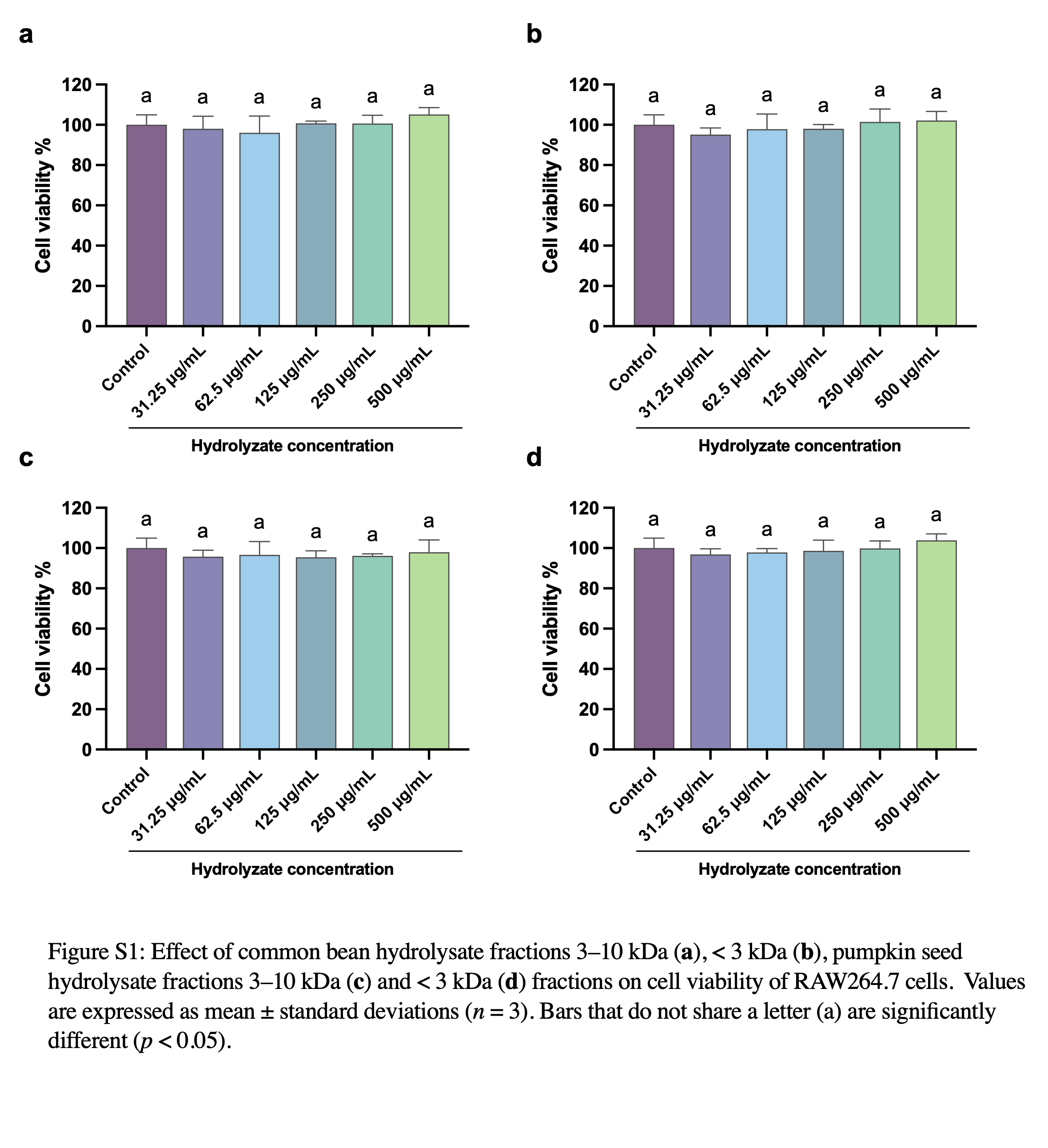

Supplement: Supplementary file 1 [file antioxidants-15-00578-s001.zip › antioxidants-4233069-supplementary.tiff]
